# Analysis and Compensation for Lateral Chromatic Aberration in a Color Coding Structured Light 3D Measurement System

**DOI:** 10.3390/s16091426

**Published:** 2016-09-03

**Authors:** Junhui Huang, Qi Xue, Zhao Wang, Jianmin Gao

**Affiliations:** 1 School of Mechanical Engineering, Xi’an Jiaotong University, Xi’an 710049, China; wangzhao@mail.xjtu.edu.cn; 2 State Key Laboratory for Manufacturing Systems Engineering, Xi’an Jiaotong University, Xi’an 710049, China; gjm@mail.xjtu.edu.cn

**Keywords:** three-dimensional sensing, color coding, chromatic aberration, compensation

## Abstract

While color-coding methods have improved the measuring efficiency of a structured light three-dimensional (3D) measurement system, they decreased the measuring accuracy significantly due to lateral chromatic aberration (LCA). In this study, the LCA in a structured light measurement system is analyzed, and a method is proposed to compensate the error caused by the LCA. Firstly, based on the projective transformation, a 3D error map of LCA is constructed in the projector images by using a flat board and comparing the image coordinates of red, green and blue circles with the coordinates of white circles at preselected sample points within the measurement volume. The 3D map consists of the errors, which are the equivalent errors caused by LCA of the camera and projector. Then in measurements, error values of LCA are calculated and compensated to correct the projector image coordinates through the 3D error map and a tri-linear interpolation method. Eventually, 3D coordinates with higher accuracy are re-calculated according to the compensated image coordinates. The effectiveness of the proposed method is verified in the following experiments.

## 1. Introduction

The structured light 3D measurement has been applied in many fields, such as industrial inspection, digital fashion, heritage conservation, reverse engineering, and aerospace [1,2,3]. The measurement efficiency is improved by color-coding methods [4,5,6,7,8]. However, the measurement accuracy is decreased by the chromatic aberration of a lens, which is inevitable. The chromatic aberration is divided into two forms, longitudinal chromatic aberration and lateral chromatic aberration (LCA). The longitudinal chromatic aberration causes image blur (the focus distance is different with different colors), while the LCA causes different positions for the object imaging with different colors although the object is in the same space position. In the structured light system, the accuracy of the position of patterns in captured image is very important for the measurement accuracy. Additionally, compared with the LCA, the influence of longitudinal chromatic aberration can be neglected. Therefore, some methods have been developed to eliminate the influence of LCA, and those methods can be divided into two categories: Hardware-based and software-based.

In hardware-based methods, some techniques of lens or device design have been developed to reduce LCA error [9,10,11,12]. Apochromatic lenses are designed to bring three wavelengths (typically the red, green, and blue) into focus in the same plane [9]. However, the residual error of off-the-shelf apochromatic lens for camera or projector is too big to be neglected. An active lens control system was studied by Willson et al. [10]. The system chromatic aberration is corrected by adjusting the distance between the image plane and lens. However, the system not only is complicated but also needs input of a priori knowledge regarding the magnification and image plane shift degree. Erwan et al. [11] designed a 3D triangulation system based on off-axis optical configuration to limit geometric and chromatic aberration, but the measurement scale is micron scale. Barone et al. [12] used a monochrome camera and replaced the color pattern with three gray patterns to avoid the chromatic aberration; however, the measuring efficiency is decreased. Generally speaking, the cost of hardware-based methods is much higher than software-based methods.

As alternatives to the hardware-based methods, algorithms are proposed to reduce the effect of LCA [13,14,15,16,17,18,19,20]. Some of them are based on the image warping method. For example, Chung et al. [13] detected the edge areas, which affected by chromatic aberration, then the color of the pixel in the areas were adjusted by image warping. Lluis-Gomez et al. [14] proposed a system based on the color intensity of the red and the blue channels correction to the green channel as a reference. Chang et al. [15] proposed a false color filter technique to eliminate LCA phenomenon in an image. Korneliussen et al. [16] proposed a post-demosaicking correction for chromatic aberration based on pixel resampling and highpass replication. Although the influence of chromatic aberration is removed by employing these image-warping methods, the pattern location is not accurate. Therefore some other researchers proposed methods to calibrate and compensate the location error. Zhang et al. [17] aligned different color channels by a checker board. The checker board was used to compute separate 2D homographies to align the color channels, but some residual misalignments were still observed because the chromatic aberration of projector was ignored. Practically, the chromatic aberration of projector is more obvious. Considering the LCA of projector, Pagès et al. [18] employed a white flat panel, which is set in front of the camera and the projector to calibrate the LCA of the system. Three patterns, which include red, green and blue fringes, respectively, were projected on to the panel and captured by the camera. The median value of relative positions of different color fringes was calculated for every scan line. The median values were used to compensate the difference of the positions between the green channel and the other two channels. However, the error caused by LCA varies with the position in the captured image, which is not considered in their study. Zhang et al. [19] proposed a method based on fringes projection. Three phase maps from different color channels were calculated and the difference of phase maps from different color channels are calculated. Based on the phase maps, a linear model between the phase difference and the position in captured image is established. Then, the phase difference caused by chromatic aberration was compensated according to the model. However, that method is just applies to the optimum multiple-fringe selection method and not suitable for general fringe projection systems. Recently, Li et al. [20] corrected the color crosstalk, as well as LCA of a color fringe projection system, applying color image segmentation and color intensity linear interpolation technique, but that method was just used for the phase reconstruction method and not applicable for the fringe encoding structured light measurement system in this study [21]. Thus, it needs an LCA correction method for fringe encoding structured light measurement system, which could achieve higher accuracy.

In this paper, an LCA error compensation method based on projective transformation is proposed. It is different from the previous LCA correction methods. It more emphasizes the location accuracy of patterns, and the location errors caused by LCA are corrected in the projector image plane instead of the camera image plane. The distance from the system to the measured point, which also influences the value of LCA, is considered. Moreover, it is applicable for the fringe encoding structured light measurement system. Thus, this study provides a general LCA error correction method and it could improve the measurement accuracy of color fringes projection systems. The following section analyzes the lateral chromatic aberration in structured light system first. Section 3 describes the principle and process of the LCA compensation method. The experiments are presented in Section 4, followed by conclusions.

## 2. Lateral Chromatic Aberration of a Structured Light System

**Figure 1:**
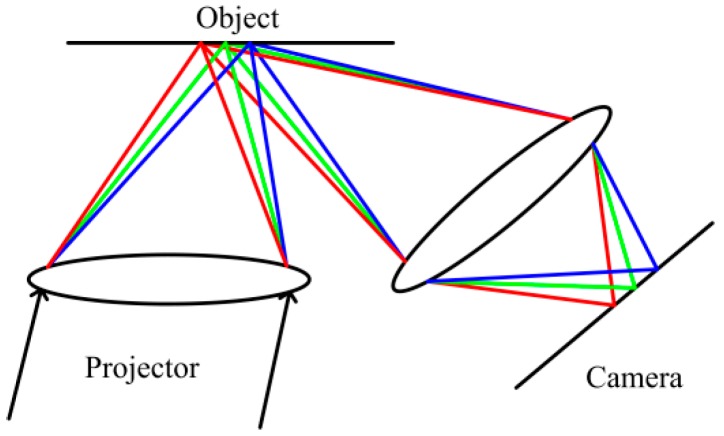
Lateral chromatic aberration (LCA) in structured light system.

A typical structured light system is composed of a camera and a projector. There are two group lenses, which belong to the camera and the projector respectively. The schematic diagram of LCA in structured light system is shown in Figure 1, which shows the LCA appears after the light goes through the projection lens.

**Figure 2:**
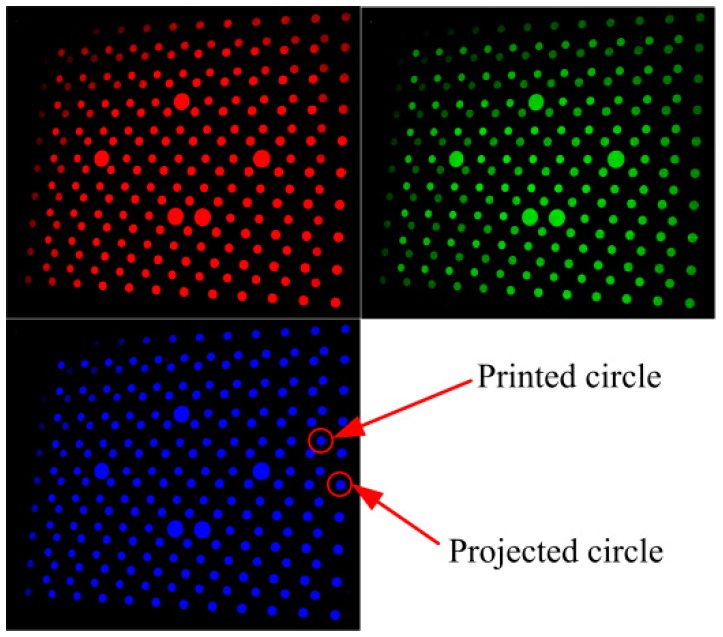
Captured color circles images.

**Figure 3:**
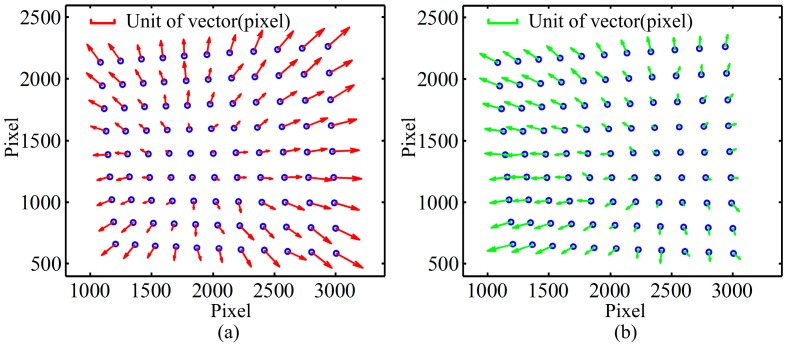
The distances between different printed color circles: (**a**) the relative distances from the red printed circles to the blue ones; (**b**) the relative distances from the green printed circles to the blue ones.

To observe the distribution of the error caused by LCA, three patterns including red, green and blue circle array are projected onto a black calibration board with white circle array respectively. The images are shown in Figure 2, which are captured by a camera. Each projection pattern contains 10 × 12 circle array, and there is a printed white circle array (9 × 11) on the calibration board. Therefore two types of circles are observed in captured images, namely, the projection and the printed ones. The centers of all circles are detected by the method proposed by Da [22]. The LCA of the camera is analyzed firstly with printed circles. Practically, any color can be chosen as the reference for calculation of the distance between different colors because white will be chosen as the base color in the following proposed method. To clearly describe the location difference of patterns between different colors, the blue is adopted as the reference because it could enlarge the calculation distances and has a strong anti-interference ability of the optical environment. Then the distances between the red and blue printed circles in corresponding positions are calculated by Equation (1), and these distances are shown as vectors in Figure 3a. Figure 3b shows the distance between the green and the blue circles. The overall image resolution is 4016 × 2688. From the vector images, it can be seen that the errors are not constant. The errors in the middle of image are small, while in the corner are relatively big. Additionally, the vectors of errors in Figure 3a,b point outward. In addition, it should be noted that the place of the minimum value is changed for different colors, for example, the place of the minimum value for the R-B is biased toward left side, while the place of the minimum value for the G-B is biased toward right side, as shown in Figure 3b. The reason would be caused by the optical system configuration of apochromatic lenses for eliminating chromatic aberration.

(1) $$d_{C}=C_{r(g)}-C_{b}$$

where ***d****_C_* represents the distance from the red or green printed circle to the blue printed circle in a corresponding position; ***C****_r(g)_* represents the camera image coordinates of the red or green printed circle center, and ***C****_b_* represents the camera image coordinates of the blue printed circle center.

**Figure 4:**
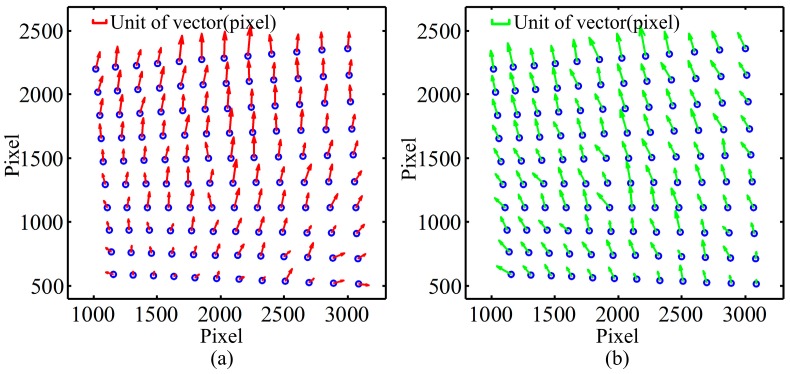
The distances between different projection color circles: (**a**) the relative distances from the red projection circles to the blue ones; (**b**) the relative distances from the green projection circles to the blue ones.

The same analysis has been conducted on projection circles, and the results are shown in Figure 4. Compared with Figure 3, the max values of the norm of vectors in Figure 4a,b are 3~4 times bigger than in Figure 3. Although the errors in Figure 4 contain both the LCA of projector and camera, the smallest vector is not in the center but in the bottom of the image and the directions of vectors are upwards as shown in Figure 4. Since projector is an image-amplifying device, the error of chromatic aberration is more obvious. In addition, the image of most commercial projectors is offset for convenience of installation and practical viewing, which results in the projection image being asymmetric with respect to the axis of projector lens. Thus, the distributions of errors are different, and the LCA errors caused by projector are much larger than those caused by camera. Therefore, the distribution of LCA error is mainly determined by lens of projector.

**Figure 5:**
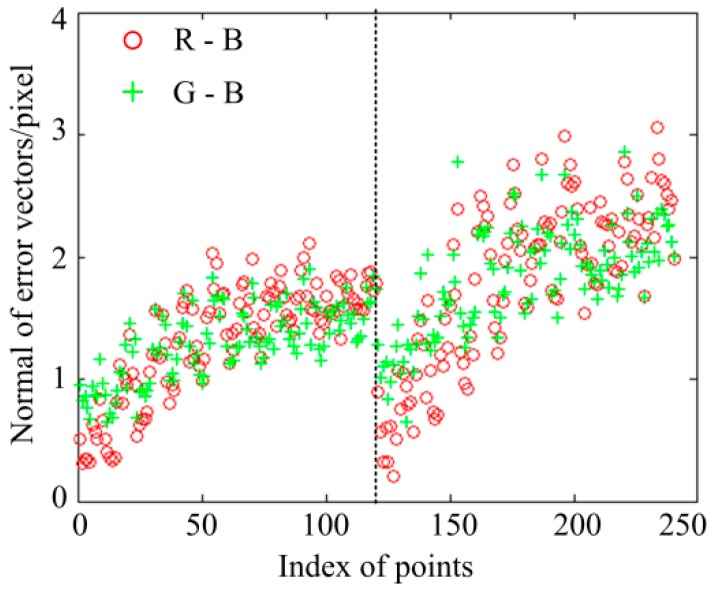
The normal of the distance vectors in tow positions.

Furthermore, the calibration board is moved to another position by a one-dimensional (1D) translation stage. The distance between the two positions is 50 mm. The identical analysis is repeated in the new position, and the magnitudes of all vectors in the two positions are shown in Figure 5. Data on the left of the imaginary line belong to the first position, while data on the right belong to the second position. In the first position, the mean and standard deviation of the R-B errors are 1.3272 pixels and 0.4624 pixels, respectively, and the mean and standard deviation of the G-B errors are 1.3008 pixels and 0.2999 pixels, respectively; while in the second position, the mean and standard deviation of the R-B errors are 1.8109 pixels and 0.6772 pixels, respectively, and the mean and standard deviation of the G-B errors are 1.8196 pixels and 0.4358 pixels, respectively. It shows the LCA errors vary in different positions. This is because the aberration chromatic error also varies with the distance between the measurement system and the measurement objects besides of the position of projection patterns.

According to the analysis above, LCA error introduced by projector is much bigger than the error introduced by camera. The LCA error distribution of projection patterns is mainly affected by lens of the projector, and the LCA error varies with the position in the image plane and distance from the measurement system to the measured object. Thus, the LCA errors are more reasonable to be corrected in the projector image plane rather than the camera image plane.

## 3. Principle and Analysis of the LCA Compensation Method

Essentially, the value of the total aberration is determined by the lens of projector and lens of camera, that is, the position of light passing through the lens. Firstly, the influence of LCA generated by the lens of projector can be described by the coordinates in the projector image plane, namely, it is determined by the position of the projection light through the projector lens. Then, the LCA becomes larger with the increase of distance, so the degree of the LCA in the object space can be expressed by that distance. Subsequently, the LCA in the object space is imaged by a camera. However, as described above, although the lens of camera also brings aberration, the amount is much lower than that of the projector and the LCA of camera can also be compensated in the projector image plane as a pre-correction. Therefore, the value of the LCA is mainly affected by the coordinates in the projector image plane and the distance between the projector and the object. In this section a 3D error map is established for the LCA compensation and the input of the error map are the two factors mentioned above.

### 3.1. Coordinate Mapping of Projection Patterns

In structured light system only camera can record error caused by LCA. However, the distribution of LCA error is mainly determined by the projector, as mentioned earlier. Therefore, correction of the chromatic aberration is more appropriate in the projector image plane, and a method is needed to transfer the error in camera image to projector image.

**Figure 6:**
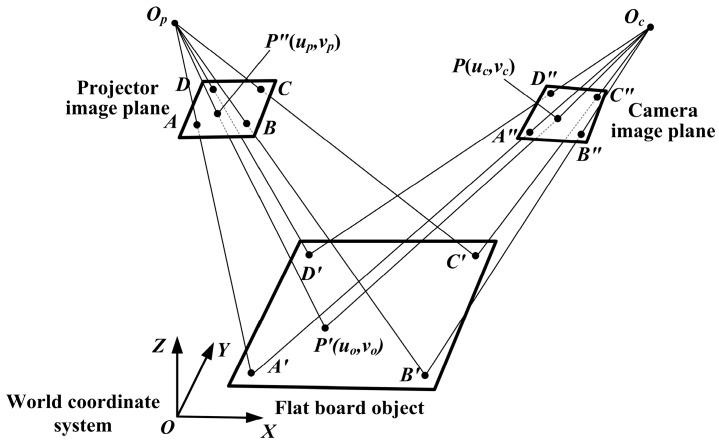
Planar projective transformation between projector and camera image planes.

The planar projective transformation can establish the relationship between them [21]. That is, the coordinates of any point *P* in camera image plane can be transformed to projector image plane by the following equation:

(2) $$\left[u_{p},v_{p},1]=M_{cp}[u_{c},v_{c},1\right]$$

where (*u_c_*,*v_c_*,1) and (*u_p_*,*v_p_*,1) are the homogeneous coordinates of *P* and *P*″ as shown in Figure 6. *M_cp_* is a 3 × 3 transformation matrix from camera image plane to projector image plane, which is solved by the points *A*, *B*, *C*, *D*, *A*″, *B*″, *C*″ and *D*″ according to least square method.

**Figure 7:**
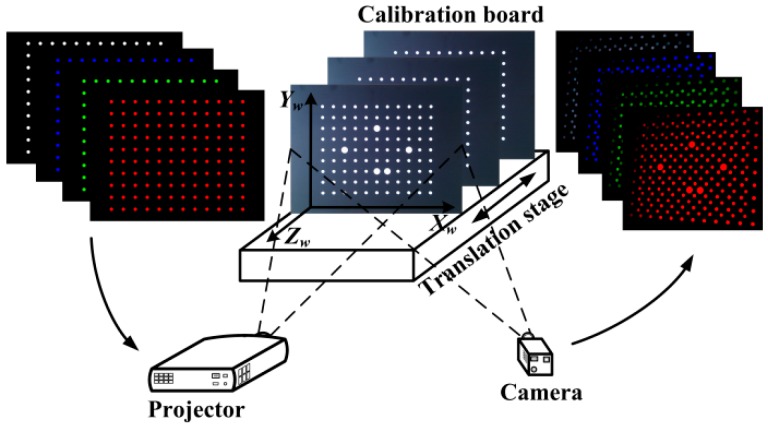
Set up of constructing a 3D LCA error map.

That 3D error map is constructed with the system calibration, but only adopts the projection patterns because the projection patterns contain both LCA errors of projector and camera. In system calibration [21], a flat board is installed on a 1D translation stage, and it can be moved within the measurement area. In the world coordinate system (the plane of the board is defined as the *X*-*Y* plane and *Z* axil is perpendicular to that plane and points to the projector, as shown in Figure 6), *z* coordinates of the points on the flat board are the constant when the board is in a certain position. At each position, it is supposed that there are four colors in color-coding patterns, which are white, red, green and blue. The four patterns (white, red, green and blue circle arrays) are projected onto the calibration board and captured by a camera in sequence, as shown in Figure 7. Those circle arrays in projection patterns cover the measurement area. The centers of the circles in the captured image are extracted and the white circles are regarded as the benchmark. Four white circle centers in the corner of image plane are selected to set up the projective coordinate systems, as shown in Figure 6. The transformation matrix *M_cp_* is solved by those four circle centers. Since the *M_cp_* varies with the depth of the calibration board, it has to be calculated for each position.

Then, all extracted circle centers of those projection patterns in the camera image plane are transferred to the projector image plane by Equation (2). There is no need to consider the perspective projection error, because only the position difference of different color circles corresponding to the same projection circle is needed. Subsequently, the flat board is moved to another position, and the patterns are projected and captured again until all acquisition positions have been completed.

### 3.2. Construction of 3D Error Map

For a projection circle, there are four transferred circle centers in the projector image plane, which are transferred from red, green, blue and white circle center in the captured image, respectively. The three directed distances from transferred red, green and blue circle centers to the transferred white circle center are calculated for each projection circle by:

(3) $$\left\{ \begin{array}{l} drw_{i}=pr_{i}(u_{p},v_{p})-pw_{i}(u_{p},v_{p})\\ dgw_{i}=pg_{i}(u_{p},v_{p})-pw_{i}(u_{p},v_{p})\\ dbw_{i}=pb_{i}(u_{p},v_{p})-pw_{i}(u_{p},v_{p}) \end{array}\right.$$

where ***d****rw_i_*, ***d****gw_i_* and ***d****bw_i_* represent the distance from transferred red, green and blue circle center to the transferred white circle center of *i*-th projection point, respectively; *p**r**_i_*(*u_p_*,*v_p_*), *p**g**_i_*(*u_p_*,*v_p_*), *p**b**_i_*(*u_p_*,*v_p_*) and *p**w**_i_*(*u_p_*,*v_p_*) represent the projector image coordinates of circle centers of *i*-th projection point in red, green, blue and white patterns after projective transformation, respectively. Those directed distances are the equivalent errors caused by LCA in projector image.

**Figure 8:**
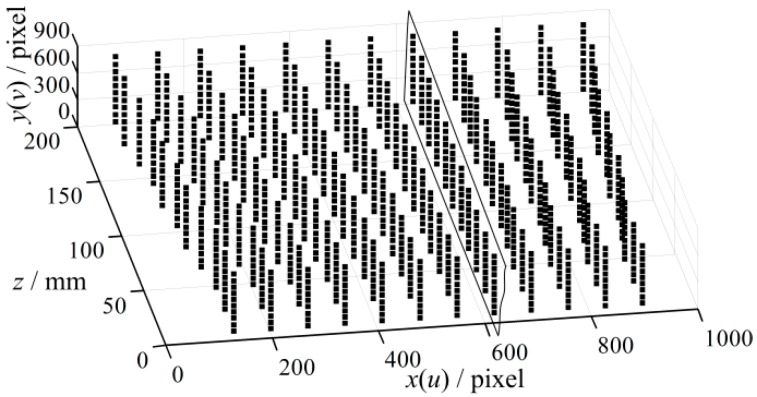
Distribution of the projection sampled points in CSEM.

**Figure 9:**
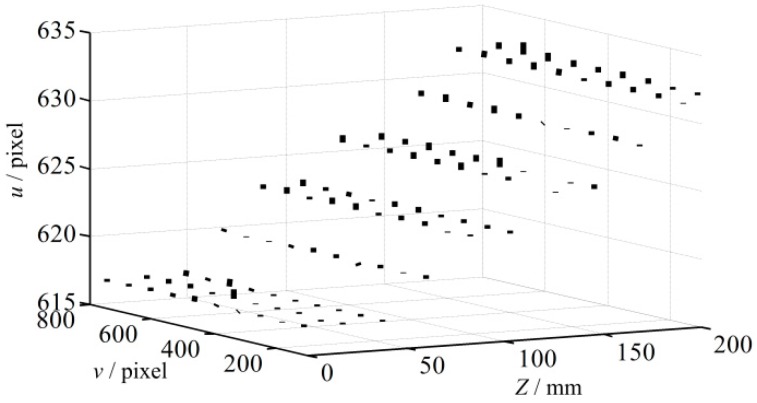
Blue error map of the slice in Figure 8.

To construct the 3D error map, 1320 projection points are preselected and the LCA errors at these points are determined. These points are arranged nearly uniformly, and a Coordinate System of the 3D Error Map is established, which is named as CSEM. The *u* and *v* axis of projector image are defined as *x* and *y* axis, and *z* axis of the measured point is defined as *z* axis, as shown in Figure 8. The value of the point is defined as the LCA error along *u* axis or *v* axis in projector image because the error is a vector. In addition, the errors of blue pattern at every point in the rectangle slice in Figure 8 are shown in Figure 9. It is shown that the maximum error can reach approximately 1 pixel after transforming onto the projector image plane. Finally, the LCA errors of every point, their corresponding projector image coordinates (*u_p_*, *v_p_*), *z* coordinates in world coordinate system and pattern colors are stored as the compensation dataset for future use.

### 3.3. Compensation of LCA Error

**Figure 10:**
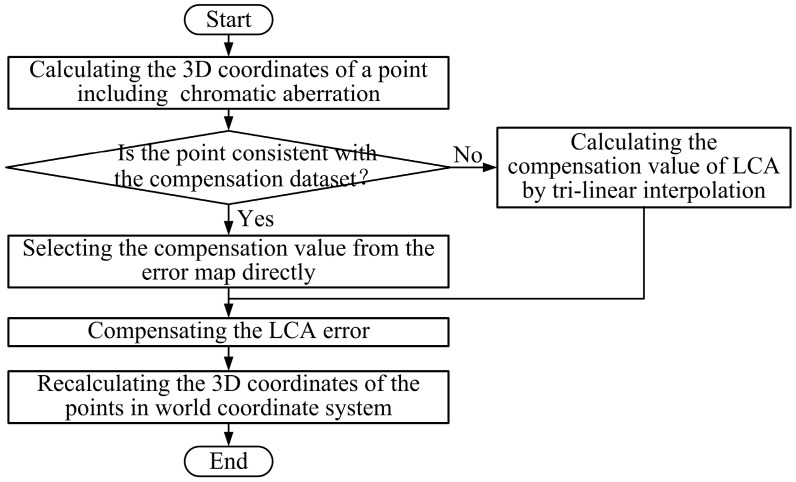
Flow chart of LCA error compensation.

After construction of the 3D error map, the LCA errors need to be compensated. The error map only includes errors for compensation in several special positions, while the chromatic aberration error is continuous. A large number of experiments show that the LCA error varies slowly in the measurement space. Therefore, a tri-linear interpolation method is used to calculate the error values, which are not included in the error map. The flow chart of the error compensation for any measured point is shown in Figure 10, and the process of error compensation is described below.

**Figure 11:**
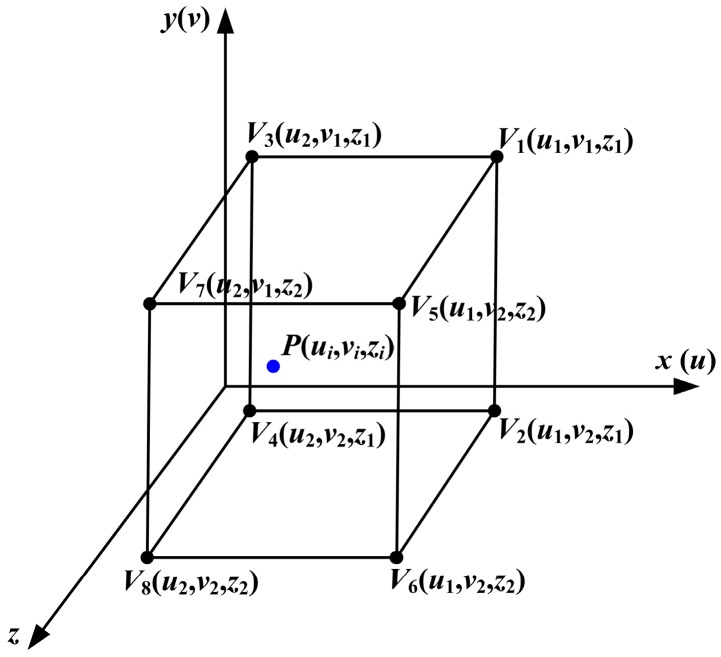
A construction hexahedron enclosing the measured point in the CSEM.

(1) Calculation of the 3D coordinates of a measured point including chromatic aberration. For each measured point, the *z* coordinate needs to be calculated for compensation, in addition to its projector image coordinates and color of projection pattern.

(2) Determination of the compensation value from the 3D error map. Firstly, we check whether the measured point is coincident with the preselected sample points in the compensation dataset, if it is, its LCA error is the error value of the matching sample point in the 3D error map; then turn to (4). If not, turn to (3).

(3) Calculation of the compensation value of LCA by tri-linear interpolation. The errors, which are not included in the compensation dataset, are calculated by tri-linear interpolation according to the 3D error map. First, the eight nearest points around the measured point are adopted as vertexes to construct a hexahedron, as shown in Figure 11. Because the circle array in the projection pattern is approximately equal interval, as shown in Figure 8, the *u* coordinates of the circles in the same column are equal and the *v* coordinates of the circles in the same row are equal as well. The interval between two slices in *z* direction is also a constant. Then, the LCA error of the measured point is calculated with the eight points by Equation (4).

(4) $$\begin{array}{cc} V_{p}= & \frac{\left(V_{1}|v_{p}-v_{1}|+V_{2}|v_{p}-v_{2}|)|u_{p}-u_{1}|+(V_{3}|v_{p}-v_{1}|+V_{4}|v_{p}-v_{2}|)|u_{p}-u_{2}\right|}{\left|u_{2}-u_{1}||z_{2}-z_{1}||v_{2}-v_{1}\right|}+\\ & \frac{\left(V_{5}|v_{p}-v_{3}|+V_{6}|v_{p}-v_{4}|)|u_{p}-u_{3}|+(V_{7}|v_{p}-v_{3}|+V_{8}|v_{p}-v_{4}|)|u_{p}-u_{4}\right|}{\left|u_{4}-u_{3}||z_{2}-z_{1}||u_{4}-u_{3}\right|} \end{array}$$

where *V*_1_(*u*_1_,*v*_1_,*z*_1_), *V*_2_(*u*_1_,*v*_2_,*z*_1_), *V*_3_(*u*_2_,*v*_1_,*z*_1_), *V*_4_(*u*_2_,*v*_2_,z_1_), *V*_5_(*u*_3_,*v*_3_,*z*_2_), *V*_6_(*u*_3_,*v*_4_,*z*_2_), *V*_7_(*u*_4_,*v*_3_,z_2_) and *V*_8_(*u*_4_,*v*_4_,*z*_2_) represent the LCA error values of vertexes of the hexahedron.

(4) Compensation of the LCA error. The projector image coordinates of the measured point are corrected according to the LCA error obtained in steps (2) or (3). The correction equation is:

(5) $$\left\{ \begin{array}{l} u_{c}=u_{i}+e_{c\_u}\\ v_{c}=v_{i}+e_{c\_v} \end{array}\right.$$

where (*u_c_*, *v_c_*) are the corrected projector image coordinates; (*u_i_*, *v_i_*) are the original coordinates of the corresponding projection pattern in projector image plane; *e_c_u_* and *e_c_v_* are the compensation values of the corresponding measured point in u and v directions.

(5) Recalculation of the 3D coordinates of the measured points in the world coordinate system. After compensation of the LCA error, the 3D coordinates of measured point are recalculated using the corrected projector image coordinates.

### 3.4. Analysis of Influence Factors

The precision of 3D error map is influenced by the projective transformation, while the accuracy of the projective transformation matrix *M_cp_* is affected by some other factors, such as lens distortion, precision of the circle detection, as well as flatness of the calibration board. The circle detection method proposed in Reference [22] is used, and the detection accuracy is 0.04 pixels. The flatness of the calibration board is less than 30 µm (corresponding to about 0.2 pixels in our system). These two factors are much smaller than the LCA error, which can be ignored. Lens distortion is the main factor causing error in projective transformation matrix *M_cp_*, which is discussed in this section.

**Figure 12:**
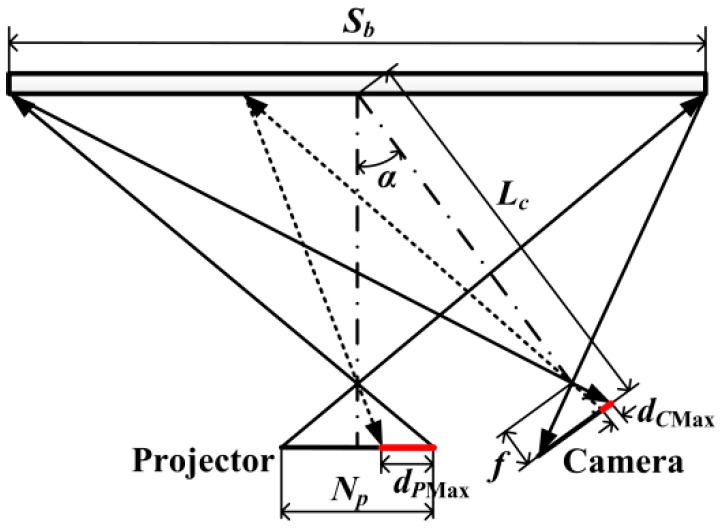
The model for calculating *d_P_*_Max_.

The sketch of system for analysis is shown in Figure 12. The max error introduced by the projective transformation is calculated for analyzing its influence. The procedures of analysis are illustrated below:

Step 1. Let the max error caused by LCA in camera image plane be *d_C_*_Max_, which is obtained by Equation (1).

Step 2. Transform the *d_C_*_Max_ to projector image plane, and let it be *d_P_*_Max_, as shown in Figure 12. The *d_P_*_Max_ is considered to be in the case of reaching the maximum value.

(6) $$\left\{ \begin{array}{l} d_{P\mathrm{Max}}=N_{p}s_{p}\cdot (\frac{1}{2}-\frac{S_{x}}{S_{b}})\\ S_{x}=\frac{L_{c}-f}{f-S_{c}\tan\alpha }\cdot d\\ d=\frac{S_{c}[f-(S_{c}-d_{C\mathrm{Max}}s_{c})\tan\alpha ]-d_{C\mathrm{Max}}s_{c}f}{[f-(S_{c}-d_{C\mathrm{Max}}s_{c})\tan\alpha ]\cos\alpha }\\ S_{c}=\frac{S_{b}f\cos\alpha }{S_{b}\tan\alpha +2(L_{c}-f)} \end{array}\right.$$

where *S_b_* is the projection range of the projector on calibration board, unit: mm; *N_P_* is the total number of pixels of projector along the vector direction of *d_C_*_Max_, unit: pixel; *s_p_* and *s_c_* are the scale parameters of projector and camera respectively, unit: mm/pixel; *L_c_* is the distance between the center of calibration board and the camera lens; *f* is the focal length of the camera lens; *α* is the angle between the direction of projection and capture; *S_x_*, *d* and *S_c_* are the intermediate variables for calculation.

Step 3. Calculate the maximum difference of projective transformation errors between two adjacent points. The *M_cp_* is calculated by using four circles in the corner, which is shown in Figure 6. The other circles’ coordinates are transferred into the projector image. The differences between the transferred coordinates and the ideal coordinates corresponding to the same projection circles are calculated, which are regarded as the projective transformation errors. In addition, two quartic polynomials are obtained by fitting the differences and their corresponding coordinates in *u* and *v* directions, respectively [21]. Then the differences of projective transformation errors between two adjacent points are calculated. The interval between the two adjacent points is *d_P_*_Max_. And the maximum value of them is selected, which is recorded as *D_Pu_*_Max_ and *D_Pv_*_Max_ in *u* and *v* directions respectively. Subsequently, the max error *D_P_*_Max_, which is caused by the error of projective transformation in the case of the maximum value, is calculated according to Equation (7).

(7) $$D_{P\mathrm{Max}}=\sqrt{D_{Pu\mathrm{Max}}^{2}+D_{Pv\mathrm{Max}}^{2}}$$

In our system [21], *D_P_*_Max_ is 0.016 pixel, which is much smaller than the vector shown in Figure 9, and can be ignored. If *D_P_*_Max_ is too large to be ignored by a system, the error caused by the error of projective transformation can be compensated by the method in Reference [21].

In addition, the inclination of the calibration board is not considered in the error map construction because the inclination is essentially to change the distance between the projector and the object or the influence of the inclination of the calibration board can be converted to the distance effect. If the LCA errors under an inclination direction of the calibration board are used to compensate the general measurement data, over or under compensation would always be generated because the orientation of a measured object may be any directions and opposite to the pre-set inclination direction of the calibration board.

## 4. Experiments and Analysis

**Figure 13:**
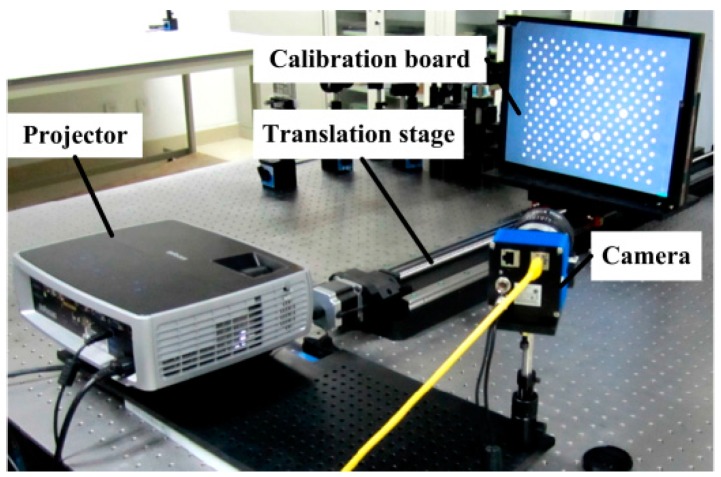
Experimental system.

The experimental system is shown in Figure 13. The system is composed of a DLP projector (InFocus IN3182, Wilsonville, OR, USA) with a resolution of 1024 × 768 and a lens of around 26 mm focal length, a CCD camera (svs 11002, Seefeld, Germany) with a resolution of 4016 × 2688 and a lens of Nikon 50 mm 1.4D, a high precision linear guide with precision of 5 µm, a calibration board with circle position precision of 15 µm and a PC. The system is calibrated according to the method in Reference [21], and the calibration scale is 400 mm × 300 mm × 180 mm.

The pattern for measurement consists of color fringes, which are the white, red, green and blue. Therefore, the patterns for constructing the 3D error map are white, red, green and blue circle array. To compare the results of the error compensation, the patterns composed of monochromatic fringes are using to measure the objects. Additionally, the method in Reference [18] is used to compensate the LCA error for comparison. In this study a 200 mm × 200 mm high precision flat board and a standard ball with a radius of 25.4 mm are measured individually.

**Figure 14:**
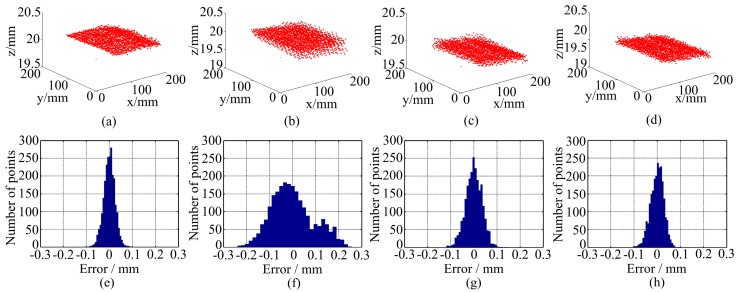
Measurement results of a flat board: 3D point clouds using (**a**) monochrome fringe; (**b**) color fringe pattern without correction; (**c**) the method in Reference [18]; (**d**) the proposed method; measurement error using (**e**) monochrome fringe; (**f**) color fringe pattern without correction; (**g**) the method in Reference [18]; (**h**) the proposed method.

Figure 14 shows the measurement results of the flat board. Figure 14a–d are the 3D point clouds and Figure 14e–h are the measurement errors. The measurement error is defined as the distance between the measured points to their fitting plane. Additionally, the max value and standard deviation of the errors are listed in Table 1. From Figure 14f it can be found that the point cloud is layered because of LCA errors. After compensation, that layered phenomenon becomes less obvious in Figure 14g,h. From Table 1, the error using monochrome fringes pattern is the smallest because no error is caused by LCA. The measurement error using color fringes pattern without correction is much bigger than the former one. The errors of the results processed by the method in Reference [18] are smaller than that of color fringe pattern without correction, while the error of the results processed by the proposed method is smaller than that by the method in Reference [18] and closer to the monochromatic fringes’ result. Both Figure 14 and Table 1 show that the proposed method is effective.

**Figure 15:**
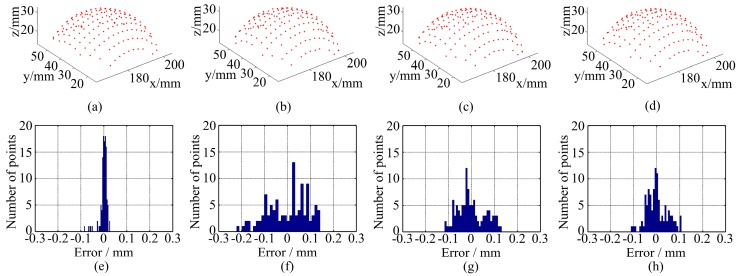
Measurement results of a standard ball: 3D point clouds using (**a**) monochrome fringe; (**b**) color fringe pattern without correction; (**c**) the method in Reference [18]; (**d**) the proposed method; measurement error using (**e**) monochrome fringe; (**f**) color fringe pattern without correction; (**g**) the method in Reference [18]; (**h**) the proposed method.

Figure 15 shows the measurement results of a standard ball and the statistics are shown in Table 2. The measurement error is defined as the distance from the data point to the spherical surface which is obtained by fitting the data to a sphere surface according to least square method. The error of radius is defined as the difference between the fitting radius and the real radius, which is obtained by a coordinates measuring machine (CMM). Figure 15 and Table 2 also show some conclusions like the measurement of the plane. The LCA has more influence on the measurement error than the radius error. In other words, both the two methods reduce the measurement error, other than the radius error, because the radius error mainly caused by noise is not reduced in captured image.

## 5. Conclusions

In this study, the error caused by chromatic aberration in structured light measurement system is analyzed. It shows that the LCA error is mainly caused by projector and varies with the location of the object for measurement. Then, a 3D error map is constructed based on a projective transformation in the projector image plane for LCA error compensation. Its input parameters are the image coordinates of a measured point in the projector and the distance from the system to the measured point, namely, it considers the effects of both position and distance. A tri-linear interpolation method is applied to obtain the compensation values when the measured point is not consistent with the preselected sample points in the 3D error map. The factors influencing the projective transformation are analyzed and the result shows those factors have little influence on the compensation precision. Finally, experimental results on a flat board and a standard ball validate the proposed method, which could achieve higher precision closer to the monochromatic fringes’ result. Thus, it is an effective method for LCA correction applied to the fringe encoding structured light measurement system. Moreover, the proposed method could also be studied to apply to the phase reconstruction method.

## Figures and Tables

**Table 1:** Statistics of the measurement errors of a flat board.

Statistics	Monochromatic Fringes	WRGB Fringes	Results of the Method in Reference [18]	Results of the Method in This Research
Max error	0.105 mm	0.258 mm	0.142 mm	0.110 mm
Std. dev.	0.025 mm	0.092 mm	0.036 mm	0.028 mm

**Table 2:** Statistic of the measurement error of a standard ball.

Statistics	Monochromatic Fringes	WRGB Fringes	Results of the Method in Reference [18]	Results of the Method in This Research
Max error	0.124 mm	0.229 mm	0.155 mm	0.150 mm
Std. dev.	0.021 mm	0.045 mm	0.036 mm	0.027 mm
Radius error	0.100 mm	0.110 mm	0.103 mm	0.100 mm

## Abbreviations

LCA	Lateral Chromatic Aberration
3D	Three-Dimensional
1D	One-Dimensional
CMM	Coordinates Measuring Machine
CSEM	Coordinate System of the 3D Error Map
